# Importance of Using Epigenetic Nutrition and Supplements Based on Nutrigenetic Tests in Personalized Medicine

**DOI:** 10.7759/cureus.66959

**Published:** 2024-08-15

**Authors:** Gulsen Meral, Elif S Aslan, Neval Burkay, Esma Gökcen Alper Acar, Mustafa Fevzi Karagöz, Merve Özkaya, Esra Sahin, Muhammed Yunus Alp

**Affiliations:** 1 Molecular Biology and Genetics, Pediatrics, Epigenetic Coaching, Norwich, GBR; 2 Molecular Biology and Genetics, Pediatrics, Biruni University, Istanbul, TUR; 3 Molecular Biology and Genetics, Biruni University, Istanbul, TUR; 4 Nutrition, Epigenetic Coaching, Norwich, GBR; 5 Medical Biology, Karamustafa Pasa State Hospital, Amasya, TUR; 6 Nutrition and Dietetics, Faculty of Health Sciences, Gazi University, Ankara, TUR; 7 Nutrition and Dietetics, Ankara University, Ankara, TUR; 8 Nutrition and Dietetics, Istinye University, Istanbul, TUR; 9 Medical Genetics, Genoks Genetics Center, Ankara, TUR; 10 Medical Genetics, Epigenetic Coaching, Norwich, GBR

**Keywords:** nutraceuticals, epigenetics, nutrigenomics, nutrigenetic, personalised nutrition

## Abstract

Background: Nutrigenetics explores how genetic variations influence an individual's responses to nutrients, enabling personalized nutrition. As dietary supplements gain popularity, understanding genetic factors in their metabolism and effectiveness is crucial for optimal health outcomes. This study examines the role of genetic differences in the metabolism and effects of nutraceuticals, underscoring the significance of personalized nutrition within precision health. It aims to reveal how individual genetic profiles influence responses to dietary supplements, highlighting the value of nutrigenetics in optimizing health interventions. The study explores how genetic variations affect the absorption and effects of nutraceuticals, focusing on personalized supplement choices based on nutrigenetics.

Methods: Sixteen patients from an Epigenetic Coaching clinic who were using supplements such as quercetin, curcumin, green tea, and sulforaphane and reporting side effects were studied. Their clinical outcomes were analyzed in relation to their supplement choices and genetic backgrounds. The study involved five women and 11 men, including eight with autism and others with conditions like Hashimoto's thyroiditis (HT) disease and joint pain.

Results: In the study, it was observed that removing sulforaphane and sulfur-rich supplements from the diet of five patients reduced agitation. Removing sulforaphane and sulfur-rich supplements from the diet of four patients reduced clinical symptoms. Green tea caused discomfort in two patients. Responses to quercetin showed clinical differences in two patients. Anxiety and hyperactivity increased in three patients who took curcumin.

Conclusion This study highlights the importance of considering individual genetic profiles when recommending dietary supplements. The findings suggest that personalized nutrition, guided by nutrigenetic insights, can enhance the efficacy and safety of nutraceutical interventions. Tailoring supplement choices based on genetic information can lead to better health outcomes and reduced adverse effects, emphasizing the need for integrating genetic testing into nutritional planning and healthcare practices.

## Introduction

In recent years, the prevalence of non-communicable chronic diseases such as cancer, cardiovascular diseases, type 2 diabetes, and autoimmune diseases, which are the leading causes of morbidity and mortality worldwide, has been steadily increasing. The Human Genome Project (HGP), initiated to explore the genetic causes of chronic diseases between 1990 and 2003, fell short of unraveling the mysteries of our genome. Consequently, projects like the Encyclopedia of DNA Elements (ENCODE) and the International HapMap Project emerged to understand the significance of epigenetic mechanisms beyond genetics in chronic diseases [1,2]. The discovery of single-nucleotide polymorphisms (SNPs) representing human genetic diversity, mainly through the HapMap project, paved the way for genome-wide association studies (GWASs) to examine genetic variants among individuals' genomes in order to define genotype-phenotype relationships [3]. Scientific advancements using whole-genome analysis methods, including SNPs, copy number variations, and other structural variants, have facilitated a comprehensive understanding of variations in the human genome. These breakthroughs have led to the development of precision medicine, also known as personalized medicine or 4P (personalized, predictive, preventive, participative), aiming to provide more effective individually tailored preventive or therapeutic medical interventions by considering individuals' genetic characteristics, lifestyle, and environmental factors [4].

Both genetic and epigenetic factors interact with environmental factors, with nutrition being one of the most influential. Nutrition not only interacts with our genetic sensitivity to food and nutrients but can also alter gene expression through epigenetic mechanisms. The epigenetic effects of nutrients have gained significant importance in preventing, alleviating, and treating many diseases [5]. Epigenetics regulates gene expression without altering the coding sequence of DNA (deoxyribonucleic acid), determining how and when specific genes will be turned on or off [6]. The comprehensive collection of epigenetic events is referred to as the epigenome, encompassing chromatin structure, histone modifications, and DNA methylation patterns in various cell types and specific environmental conditions [7]. In this context, nutri-epigenetics focuses on how nutrition regulates the opening and closing of particular genes. Nutri-epigenomics, on the other hand, involves analyzing the effects on global gene expression that can vary between different tissues due to interactions between numerous genes and nutrition [6]. Nutrition can directly impact the expression of genes that regulate critical metabolic pathways. Nutrigenomics, the science of how nutrients and bioactive food compounds influence gene expression, explores the role of foods in gene expression [8]. Genetic variations can alter protein synthesis and the functions of synthesized proteins, thereby influencing individuals' nutritional needs and metabolism, potentially affecting the risk of chronic diseases [9]. Nutrigenetics encompasses the effects of genetic diversity on nutritional responses and nutrient function [10].

Epigenetic diversity may be affected by dietary factors. It is essential to investigate strategies that use dietary compounds to target epigenetic modifications. Sulforaphane (SFN) is a natural dietary isothiocyanate found in cruciferous vegetables, especially broccoli, Brussels sprouts, cabbage, and cauliflower [11]. Recent studies have shown that SFN influences certain cancers through epigenetic mechanisms such as microRNA (miRNA) modulation, histone deacetylase (HDAC) inhibition, and reducing the expression of DNA methyltransferase 1 (DNMT1) [12-15]. Curcumin is a yellow-orange polyphenolic compound obtained from the roots of the *Curcuma longa* plant [16-17]. Curcumin can also epigenetic regulation. The epigenetic regulatory roles of curcumin include the regulation of histone modifications via the regulation of histone acetyltransferases (HATs) and HDACs, the inhibition of DNA methyltransferases (DNMTs), regulation of miRNA, action as a DNA binding agent, and interaction with transcription factors [18-20]. Quercetin is a flavonol found in high quantities in plants, especially vegetables and fruits [21]. The epigenetic regulatory roles of quercetin include the inhibition of DNMTs and the regulation of histone modifications via the regulation of HDAC inhibition [22]. Epigallocatechin gallate (EGCG), found as a flavonoid in green tea, is the primary catechin of green tea [23]. In the context of epigenetics, EGCG exhibits anti-cancer effects by explicitly inhibiting the DNMT enzyme and promoting HDAC inhibition [24].

This study aims to investigate the effects of hereditary genetic variants on the intake and metabolism of micronutrients while highlighting the impact of patients' nutrigenetics-based epigenetic supplement preferences. Changes in clinical profiles were retrospectively presented based on whether supplements were chosen or not, considering patients' clinical history and genetics. The study evaluates the use of supplements such as SFN, curcumin, quercetin, and green tea, taking into account their effects on gene expression from a nutrigenetics-based epigenetic nutrition perspective.

## Materials and methods

This study was conducted with the approval of the Biruni University Ethics Committee under reference number 2023/78-03. The patient group was selected retrospectively from people who had nutrigenetic testing in Epigenetic Coaching. A group of 16 patients who applied to the Epigenetic Coaching between 01/01/2023 and 01/01/2024 and used quercetin, curcumin, green tea, or sulforaphane and who were experiencing side effects participated. Genetic tests designed by our team were then compared. This study was conducted at Molecular Biology and Genetics, Biruni University, Istanbul, Turkey.

Inclusion criteria for this study are as follows: individuals with various allergic diseases such as allergies and eczema, as well as autism; individuals with epigenetic autoimmune diseases; participants aged between five and 60 years; individuals without a diagnosed genetic disease; female patients not undergoing pregnancy; and patients not using autoimmune and gastrointestinal medications. Exclusion criteria for this study are as follows: patients under the age of five and over the age of 60 and female patients undergoing pregnancy use of autoimmune and gastrointestinal medications.

Genetic analysis

Genomic DNA was extracted from buccal swabs using the phenol/chloroform extraction method. The isolated DNA's quality and concentration were assessed using a NanodropTM 1000 spectrophotometer (Thermo et al., Wilmington, DE, USA) and then normalized to 50 μl/mL. The samples were analyzed on an Illumina iScan (Illumina, San Diego, CA, USA) using a custom Infinium HTS iSelect microarray. Illumina GenomeStudio V2.0.5 software was employed to determine SNP alleles [25].

## Results

This study aimed to investigate the effects of inherited genetic variants on the intake and metabolism of micronutrients and to reveal the effects of patients' nutrigenetic-based epigenetic supplement preferences. The effects of bioactive food compounds such as SFN, curcumin, quercetin, and also green tea on gene expression were taken into account with the nutrigenetic-based epigenetic nutrition approach, which covers the nutritional and genetic interaction perspective (Table 1).

**Table 1:** Effects of nutraceuticals on genes CYP1A1: cytochrome P450, family 1, subfamily A, polypeptide 1; CYP1A2: cytochrome P450, family 1, subfamily A, polypeptide 2; GST: glutathione S-transferases; COMT: catecol-O-methyltransferases; CBS A360A: cystathionine beta-synthase A360A; CBS C699T: cystathionine beta-synthase C699T

Nutraceuticals/genes	CYP1A1	CYP1A2	GST	COMT	MAO	CBS A360A	CBS C699T
Sulforaphane	Inhibit	Inhibit	Induces	Induces	-	Induces	Induces
Quercetin	Inhibit	Inhibit	Induces	Inhibit	Inhibit	-	-
Green tea	Induces	Induces	Induces	Inhibit	Inhibit	-	-
Curcumin	Inhibit	Inhibit	Induces	-	Inhibit	-	-

Retrospectively, 16 people (five female and 11 male) who felt discomfort or well after using epigenetic supplements such as quercetin, curcumin, green tea, and SFN were included in the study. Eight of the people included in the study had autism, one had an allergy, and one had a cardiovascular disease. There are different disease complaints, such as atopic dermatitis, autoimmune thyroiditis, seborrheic blepharitis, allergy-autoimmune thyroiditis (1), and ulcerative colitis (1) (Table 2).

**Table 2:** Characteristics of the participants ASD: autism spectrum disease

Patient	Age	Sex	Disease	Complaints
OO	23	Male	ASD^*^	When sulfur was ceased, agitation decreased.
TK	12	Male	Allergy, ASD	When sulfur was ceased, agitation decreased, and allergies decreased
AU	11	Male	ASD	Quercetin caused urinary incontinence, and when sulfur was ceased, agitation decreased.
HDA	29	Male	ASD	Quercetin increased anxiety.
ABÖ	8	Male	ASD	When sulfur was ceased, agitation decreased.
MAE	9	Male	ASD	Curcumin increased hyperactivity, yelling, and obsessiveness.
ZJ	10	Male	ASD	When sulfur was ceased, agitation decreased.
AM	8	Male	ASD	When sulfur was ceased, agitation decreased. Curcumin increased hyperactivity and yelling.
SSO	37	Female	Allergy	When sulfur was ceased, agitation decreased.
CD	12	Female	ASD	When sulfur was ceased, agitation decreased
EY	45	Female	Autoimmune thyroid	Increased agitation and discomfort.
OG	43	Male	Cardiovascular	Green tea caused discomfort.
YG	22	Male	Atopic dermatitis	When sulfur was ceased, agitation decreased
İA	57	Female	Allergy - autoimmune thyroid	When sulfur was ceased, agitation decreased.
YM	50	Female	Seborrheic blepharitis	When sulfur was ceased, agitation decreased.
OE	31	Male	Ulcerative colitis (UC)	Curcumin increased anxiety.

The effect of sulforaphane-sulfur-containing foods on COMT and CBS was examined based on the genotype of the cases (Table 3). While three autism patients have homozygous (rs4633-TT and rs4680- AA) genotypes in both the COMT variants (rs4633 and rs4680), one autism patient has heterozygous (rs4633-CT and rs4680-AG) genotypes in both variants. A patient suffering from joint pain was found to have a heterozygous (rs4633-CT; rs4680-AG) genotype for both COMT polymorphisms. When looking at CBS polymorphism, four autism cases carry a heterozygous (CT) genotype and one joint pain case has a heterozygous (CT) genotype (Table 3).

**Table 3:** Sulforaphane-sulfur-containing food (e.g., broccoli) usage and COMT and CBS polymorphism interactions ASD: autism spectrum disease, SFN: sulforaphane

Patient	Disease	Symptoms	COMT-rs4633	COMT-rs4680	CBS-699
OO	ASD^*^	When sulfur-SFN^** ^was ceased, agitation decreased.	TT (slow)	AA (slow)	CT (medium)
TK	ASD	When sulfur-SFN was ceased, agitation decreased.	TT (slow)	AA (slow)	CT (medium)
ABÖ	ASD	When sulfur-SFN was ceased, agitation decreased.	CT (medium)	AG (medium)	CT (medium)
ZJ	ASD	When sulfur-SFN was ceased, agitation decreased.	TT (slow)	AA (slow)	CT (medium)
SS	Joint pain	When sulfur-SFN was ceased, agitation decreased.	CT (medium)	AG (medium)	CT (medium)

The effects of SFN-sulfur-containing foods on CYP1A1, CYP1A2, GST, and CBS were examined. All cases in the table have the homozygous (TT) genotype in the CYP1A1 genetic variant. In the CYP1A2 variant, two people (1 allergy-Hashimoto and one autism patient) carry the homozygous (AA) genotype, and two people (one allergy, one eczema) carry the heterozygous (AC) genotype. While CD and YG cases have a homozygous (GG) genotype in the GSTA1 (glutathione S-transferase alpha 1) variant, IA and YM cases have a heterozygous (AG) genotype. IA and YM cases carry the homozygous (AA) genotype in the GSTP1 (glutathione S-transferase pi 1) variant. The CD case has the homozygous (GG) genotype in the GSTP1 variant, while the YG case has the heterozygous (AG) genotype. YG and İA have a homozygous (TT) genotype in the CBS 360 variant and a homozygous (CC) genotype in the CBS 699 variant. The YM case has a heterozygous CBS 360 (CT) CBS 699 (CT) genotype in both CBS variants. The CD case carries the homozygous (CC) genotype in the CBS 360 variant and the homozygous (TT) genotype in the CBS 699 variant (Table 4).

**Table 4:** Sulforaphane-sulfur-containing food (e.g., broccoli) usage and CYP1, GST, and CBS polymorphism interactions ASD: autism spectrum disease, AT: autoimmune thyroid

Patient	Disease	Symptoms						
			CYP1A1	CYP1A2	GSTA1	GSTP1	CBS360	CBS699
CD	ASD^*^	Broccoli sprouts were not tolerated well.	TT slow	AA fast	GG fast	GG slow	CC slow	TT fast
YG	Atopic dermatitis, eczema	After sulphur sources were cut, recovery was better.	TT slow	AC medium	GG fast	AG medium	TT fast	CC low
İA	Allergy- AT^**^	After sulphur sources were cut, recovery was better.	TT slow	AA fast	AG medium	AA fast	TT fast	CC low
YM	Allergy	After sulphur sources were cut, recovery was better.	TT slow	AC medium	AG medium	AA fast	CT medium	CT medium

The effects of curcumin on CYP1A1, CYP1A2, and MAO were examined. The three cases seen in the table have the genotype, homozygous (TT) for both CYP1A1 and the MAO variant. The MAE case has a homozygous (AA) genotype in the CYP1A2 variant (MAE took 100 mg of curcumin per day for one week). Case AM has a heterozygous (AC) genotype in the CYP1A2 variant (AC took 100 mg curcumin per day for four months), and case OE (OE took 500 mg curcumin per day for one month) has a homozygous (CC) genotype (Table 5).

**Table 5:** Curcumin usage and CYP1 and MAO polymorphism interactions ASD: autism spectrum disease, UC: ulcerative colitis

Patient	Disease	Symptoms	CYP1A1	CYP1A2	MAO
MAE	ASD^*^	Curcumin increased hyperactivity, yelling, and obsessiveness.	TT (slow)	AA (fast)	TT (slow)
AM	ASD	Curcumin increased hyperactivity and yelling.	TT (slow)	AC (medium)	TT (slow)
OE	UC^**^	Curcumin increased anxiety.	TT (slow)	CC (slow)	TT (slow)

The effects of quercetin on CYP1A1, CYP1A2, COMT, and MAO were examined. Homozygous (TT) slow genotype in the CYP1A1 and MAO variants was observed in two cases of autism patients. In the CYP1A2 variant, both cases carry the heterozygous (AA) fast genotype. In the AU case, both COMT variants have a homozygous (rs4680-TT and rs4633-AA) slow genotype (AU took 500 mg quercetin per day for two years). In the HDA case, heterozygous (rs4680-CT and rs4633-AG) genotypes were observed in the COMT variant (HDA took 500 mg quercetin per day for two years) (Table 6).

**Table 6:** Quercetin, CYP1, COMT, and MAO polymorphism interaction ASD: autism spectrum disease

Patient	Disease	Symptoms	CYP1A1	CYP1A2	COMT (rs4680)	COMT (rs4633)	MAO
AU	ASD*	Urinary incontinence was observed.	TT (slow)	AA (fast)	TT (slow)	AA (slow)	TT (slow)
HDA	ASD	Quercetin increased anxiety.	TT (slow)	AA (fast	CT (medium)	AG (medium)	TT (slow)

The effects of green tea on CYP1A1, CYP1A2, GST, COMT, and MAO were examined. OG and EY cases have a homozygous (TT) genotype in the CYP1A1 variant and a heterozygous (AC) genotype in the CYP1A2 variant. While the OG case carries a heterozygous (CT, AG) genotype in COMT polymorphisms, the EY case has a homozygous (CC, GG) fast genotype in COMT variants. OG case has a homozygous (GG) fast genotype in the MAO variant, while the EY case has a homozygous (TT) slow genotype in the MAO variant (EY took 400 mg EGCG per day for one month) (Table 7).

**Table 7:** Green tea interactions

Patient	Symptoms	CYP1A1	CYP1A2	COMT (rs4680)	COMT (rs4633)	MAO
OG	Disturbed	TT (slow)	AC (medium)	CT (medium)	AG (medium)	GG (fast)
EY	Disturbed	TT (slow)	AC (medium)	CC (fast)	GG (fast)	TT (slow)

## Discussion

Our study aimed to examine the use of epigenetic dietary components based on nutrigenetic test results. It is known that bioactive components such as SFN, curcumin, EGCG, and quercetin have efficacy on phase 1 and phase 2 detoxification enzymes. In this study, we observed the clinical outcomes of supplement preferences based on the status of enzyme activities related to variants in cytochrome P-450 1A (CYP1A), GST, COMT, MAO, and CBS genes. The importance of choosing supplements in clinical practice based on nutrigenetically effective epigenetic agents has been demonstrated through our cases.

Broccoli, cauliflower, cabbage, and other cruciferous vegetables are preferred in clinical nutrition due to the interaction with enzymes involved in detoxification, specifically isothiocyanates found in these vegetables. Mainly, CYP1A2, a biotransformation enzyme that activates many procarcinogens, is induced by cruciferous vegetables [26]. In addition, sulforaphane found in cruciferous vegetables is an anticarcinogenic compound. It has been demonstrated that sulforaphane inhibits the activity of CYP1A1 and CYP1A2 induced by compounds causing cancer, upregulates phase II enzymes like GSTs, and plays an inducing role in enzyme activities when added to the diet. In addition, sulforaphane, an antioxidant present in cruciferous vegetables, successfully reversed estrogen-induced epigenetic modifications and the gene silencing of COMT [13,27-29]. 

Sulforaphane, a component of isothiocyanate derived from cruciferous vegetables, releases hydrogen sulfide (H2S) in Brassicaceae. It accomplishes this through the CBS metabolic pathway, activating H2S production. SFN achieves this effect through CBS activation [30]. CBS is an enzyme that mediates H2S production using homocysteine or methionine [31]. In a study, genetic overexpression of CBS in the brain resulted in disruptions in serotonin and dopamine pathways, contributing to neuronal disorders associated with Down syndrome [32]. Increased H2S due to CBS upregulation has been observed to have an impact on mental impairment and has been indicated to affect the cognitive phenotype in Down syndrome. Overexpression of CBS was observed in the hippocampus and cortex of Down syndrome mice. Therefore, it has been suggested that CBS inhibitor-containing treatment might be preferable for improving the quality of life in individuals with Down syndrome [33]. Based on these studies, a therapeutic approach may be considered that includes CBS inhibitors and dietary or supplement options to prevent overexpression of CBS. In our study, it was observed that agitation decreased in cases carrying the T allele of the CBS 699 polymorphism (OO, TK, ABO, ZJ, and SS) when sulfur-sulforaphane-containing foods were stopped (Table 3) [34] by using reducing the consumption of sulforaphane-sulfur containing foods in these cases reduced the release of H2S. In another study, the proinflammatory effects of increased H2S due to CBS upregulation and its contribution to inflammatory pain were observed. It was noted that the elevated H2S, along with neural hyperexcitability, induced inflammation, and CBS inhibitors reduced H2S levels, thereby reversing this effect [35]. In cases with the CYP1A2 rs 762551 (AA) fast genotype (CD, YG, IA, and YM), given in Table 4, sulfur-containing foods such as broccoli and cruciferous vegetables may have increased side effects because they accelerated CYP1A2 [36-37]. Therefore, we believe that eliminating sulfur-containing foods such as broccoli and cruciferous vegetables, which increase CYP1A2 activity and upregulate CBS, may benefit patients with inflammation due to the decreased effect on CYP1A2 and the reduced H2S increase in genotypes with increased upregulation on CBS [26,35-38].

The activity of CYP1A1 and CYP1A2 in rat liver was examined to investigate the chemopreventive mechanisms mediated by turmeric and to compare the chemopreventive efficacy of curcumin, with reported inhibitory effects of curcumin [39]. In addition, according to a meta-analysis, daily doses of curcumin or turmeric did not significantly alter liver enzymes [40]. Studies relating liver damage cases with the use of food supplements containing turmeric emphasize that caution should be taken when using curcumin [41]. In our study, from a nutrigenetic perspective, it stands out that this tolerance is not the same for every individual, and the inhibitory effect of curcumin on the evaluated phase 1 enzymes should be considered. Curcumin supplementation may be carefully considered in individuals with genetic variants of CYP1A1 rs1048943 (TT), which causes slow enzyme activities [42] (Table 1). In our study, cases (MAE, AM, and OE) with TT genotype causing slow enzyme activity were observed to not benefit from curcumin use (Table 5).

Studies investigating the impact of curcumin on antioxidant and phase II metabolizing enzyme activities involved in detoxification indicate an increase in GST gene expression and enzyme activity with curcumin application. Curcumin demonstrates protective potential against chemical carcinogenesis and toxicity [43-45]. In this context, curcumin supplementation might be recommended for individuals with slow GST enzyme activity; however, considering the inhibitory effect of curcumin on CYP1A1 and CYP1A2 enzymes is essential. Curcumin, including some phenolic dietary compounds, is considered a potential source of MAO inhibitors used in the treatment of Parkinson's and other neurological disorders due to its inhibitory effect on MAO activity responsible for the metabolism of monoamine neurotransmitters crucial for neural development and function [46]. Therefore, before choosing versatile and bioactive nutraceuticals like curcumin as supplements, it is essential to assess individuals' genetic makeup. In our study, it was observed in cases (MAE, AM, and OE) with the TT genotype in the MAO rs6323, causing slow enzyme activity [47] (Table 1), that curcumin did not have a beneficial effect (Table 5).

Quercetin has been shown to inhibit the function of drug-metabolizing enzymes CYP1A1 and CYP1A2 and upregulate GST enzymes [48-50]. Quercetin's inhibition of COMT activity could lead to prolonged exposure of breast cells to E(2) and catechol estrogens, allowing more time for the carcinogenic metabolites of E(2) to act. This extended exposure could also increase oxidant stress due to the metabolic conversion to estrogen metabolites, potentially worsening E(2)-induced breast tumors in female ACI rats [51].

Quercetin has been observed to exhibit antidepressant-like effects in mice exposed to stress by preventing brain oxidative stress and restoring serotonin levels through the inhibition of MAO activity [52]. These studies suggest that the inhibitory effect of quercetin on MAO can provide benefits or harm depending on individuals' genetic makeup. The presence of the T allele in the MAO gene is associated with moderate or low MAO levels, leading to an increase in dopamine levels and contributing to certain neurodegenerative disorders [47]. In our cases, individuals with the T allele in the MAO rs6323 (AU and HDA) who used quercetin experienced increased agitation, which was thought to be related to this condition (Table 6). Another study suggests that individuals with slowing alleles in the COMT rs4680-AA [53] (Table 1) and COMT rs4633-TT [54] (Table 1) as observed in our negatively affected cases (HDA-AU) may experience increased sensitivity to anxiety and stimuli, potentially explaining such effects (Table 6) [55].

Quercetin has been shown to be protective against urinary incontinence in one study [56]. However, in our study, the use of quercetin did not benefit the case AU, who had slow genotypes in CYP1A1 rs1048943-TT [42], COMT rs4680 -AA [53], rs4633-TT [54] and MAO rs6323-TT [47] (Table 1), and urinary incontinence was observed after quercetin use (Table 6). These results suggest the importance of genetic tests in personalized supplement selection for individuals.

Green tea plays a regulatory role as a chemo-preventive agent in both phase 1 and phase 2 conjugating enzymes. It has been reported that green tea increases glutathione levels, reduces oxidative stress, modulates carcinogen metabolism, and induces an increase in CYP1A1, CYP1A2, and GST expressions [57-58]. On the contrary, some authors demonstrated the activatory effect of green tea on the cytochrome oxidases and suggested that the effect of green tea on CYP1A1 and CYP1A2 enzyme activity is differentiated with caffeine compound, sex, and pre-existing level of enzyme expression [57,59-60].

The polyphenols in green tea, particularly EGCG, are significantly affected by the anticancer activities of COMT activity. In addition, flavonoids inhibit the COMT enzyme, reducing the detoxification of endogenous catechol estrogens [61]. The enzyme COMT is involved in metabolizing EGCG through O-methylation. Lower COMT activity may heighten sensitivity to EGCG-induced hepatotoxicity, given COMT's vital role in shielding cells from oxidative stress and liver damage. This study indicates that EGCG acts both as a substrate for and a significant inhibitor of human liver cytosolic COMT, displaying inhibitory effects on this enzyme. The COMT gene, crucial in metabolizing catecholamines, influences norepinephrine and dopamine levels. People with the slow A allele of the COMT gene (rs4680 polymorphism) may face a higher risk of anxiety, neuropsychiatric issues, and heightened sensitivity to environmental toxins [55,62-63]. For instance, OG, carrying the COMT rs4680-AG genotype, experienced increased agitation with green tea consumption, as shown in the findings. Thus, individuals with reduced COMT activity are advised to be cautious with green tea intake. The inhibition of catechol-O-methyltransferase by green tea's epigallocatechin gallate not only contributes to its health benefits but also helps prolong norepinephrine's action in the synaptic gap [64]. In a study investigating MAO enzyme inhibition, green tea was found to have the highest inhibition level of MAO forms among teas [65]. In this case, individuals with slow enzyme activity [47] (Table 1) in the MAO rs6343 may need to avoid consuming green tea. Similar to our study, in Table 7, it was observed that the consumption of green tea increased agitation and discomfort in the case of EY, who had the TT genotype, causing slow enzyme activity [47] (Table 1) in the MAO rs6343.

In our study, considering the approach that green tea's inducing effect, due to slow alleles in the CYP1A1 rs1048943-T [42] (Table 1) and CYP1A2 762551-C [38] (Table 1) genes in cases OG and EY, could lead to an increase in intermediate metabolites, we can hypothesize that it may cause a disturbing effect in these patients (Table 7). In our study, we wanted to highlight the clinical responses regarding nutrigenetic differences in the use of epigenetic nutrition and supplements, especially for the purpose of clinical success and minimizing the side effects of supplements. Although the number of cases is limited, we believe that our study will shed light on personalized supplement choices.

## Conclusions

This comprehensive exploration of the interactions between dietary components and enzymatic processes highlights the intricate relationship between genetics, nutrition, and health outcomes. The individualized responses to cruciferous vegetables, sulforaphane, quercetin, green tea, and curcumin underscore the significance of tailoring nutritional strategies based on genetic profiles. As we delve deeper into the field of nutrigenomics, it becomes increasingly evident that understanding an individual's genetic makeup is crucial for optimizing the potential benefits of dietary choices and supplements while minimizing potential risks.
